# Physical Therapy and Rehabilitation Approaches in Patients with Carpal Tunnel Syndrome

**DOI:** 10.7759/cureus.7171

**Published:** 2020-03-03

**Authors:** Aicha Zaralieva, Georgi P Georgiev, Vesselin Karabinov, Alexandar Iliev, Assen Aleksiev

**Affiliations:** 1 Physical Medicine and Rehabilitation, Queen Giovanna Hospital, Sofia, BGR; 2 Orthopaedics and Traumatology, University Hospital Queen Giovanna, Sofia, BGR; 3 Neurology, National Cardiology Hospital, Sofia, BGR; 4 Anatomy, Histology and Embryology, Medical University of Sofia, Sofia, BGR; 5 Physical Medicine and Rehabilitation, Medical Univeristy of Sofia, Sofia, BGR; 6 Physical Medicine and Rehabilitation, Aleksandrovska University Hospital, Sofia, BGR

**Keywords:** carpal tunnel syndrome, physical therapy, treatment algorithm

## Abstract

Physical therapy involves a set of factors and methods that affect the biological processes in the body. It is widely used, relatively inexpensive, non-invasive, and easy to apply. Physiotherapy is also used in the treatment of patients with carpal tunnel syndrome (CTS). This syndrome represents the most common compressive mononeuropathy of the upper limb and leads to significant disability. Hence, its successful treatment leads to significant benefits for both the patient and society. There is no established algorithm for the use of physical therapy in these patients. In this publication, we present the physiotherapeutic methods used for the treatment of CTS both before and after surgical treatment.

## Introduction and background

Physical therapy or physiotherapy (derived from the Greek word fysis, meaning nature, and therapia, meaning treatment) combines a variety of factors aimed at the prevention and treatment of diseases by means of natural factors (sun, sea, healing mud, water, movement) and preformed factors (electric current, ultrasound, artificial light including laser rays, magnetic field, etc.). Their action reduces pain, stimulates restoration processes, increases range of motion, activates immune mechanisms, and improves biochemical performance. Compared to other therapies the physical therapy is cheaper, non-invasive, and easy to apply (Paper presentation: Troev T, Zaralieva A, Lutskanova S. The Application of Physical Medicine in Medical Practice. Second International Scientific and Practical Conference: "Nature, Forest, Society. Nature and Habitats, Human activity and Hunting, Alternative and Hunting tourism. Problems and Interconnection. BLRS in 2014"; 2014).

Carpal tunnel syndrome (CTS) is the most common compressive mononeuropathy of the upper limb [1-5]. CTS affects 4-6% of the population [6]. Treatment of CTS should be started as early as possible. In a large percentage of cases where anatomical abnormalities of the carpal canal are not present, the functionality of the affected hand can be restored by appropriate physiotherapy rehabilitation programs [6-12,13,14].

An optimal combination of physical factors and kinesiotherapy is sought, depending on the disease stage, the severity of the symptoms, the proven objective changes, the individual characteristics of the patient, and accompanying diseases. Typically, a 10-day course of physiotherapy is carried out, which can be repeated several times after a break of 2-4 weeks.

## Review

Physical factors involved in the treatment of CTS

*Therapeutic Courses Usually Include A Combination Of The Following Physical Factors* ​​​​

**Figure 1:**
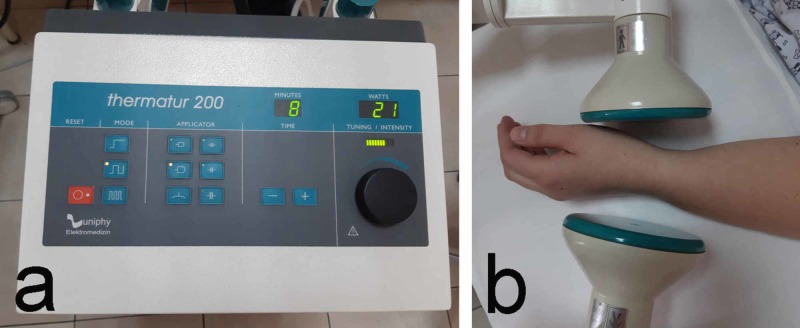
Ultra high frequency therapy a: device; b: application technique

1. Exogenous heat by means of paraffin, or endogenous heat by ultra high frequency therapy (UHFT) is used based on the disease stage, the severity of the symptoms, the selectivity of the treated tissues according to their water percentage and the patient's individual tolerance (Figure 1).

Paraffin is applied at a temperature of about 50 °C for 15-20 minutes on the anatomical projection of the carpal canal and the palm. UHFT is applied with athermic doses (lack of heat sensation) to oligothermic doses (minimal to moderate heat sensation). The capacitive electrodes are used in order to heat selectively the tissues with a lower water percentage. The distance from the patient's skin to the electrodes is about 2-3 cm. The duration of the procedure is 8-10 minutes. The therapeutic course consists of 10 procedures. Thermal procedures are used for analgesia, reduction of paresthesia, stiffness, and improvement of nervous conduction and trophy. They could also be used as an introductory procedure for subsequent ultraphonophoresis. Another heat factor alternative is the targeted radiofrequency therapy (Tekar therapy); it is a method where high-frequency electromagnetic waves cause warming in depth. The principle of this therapy is also known as long-wave diathermy. It has an anti-inflammatory effect and accelerates the regeneration of tissues. The devices combine two modes of operation: capacitive (460 kHz) and resistive (460 kHz) [15].

**Figure 2:**
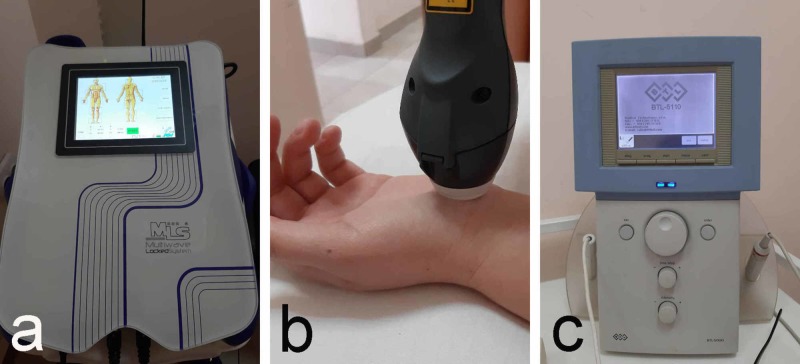
Laser therapy a: device (MLS Laser); b: application technique; c: device for low-intensity laser therapy

**Figure 3:**
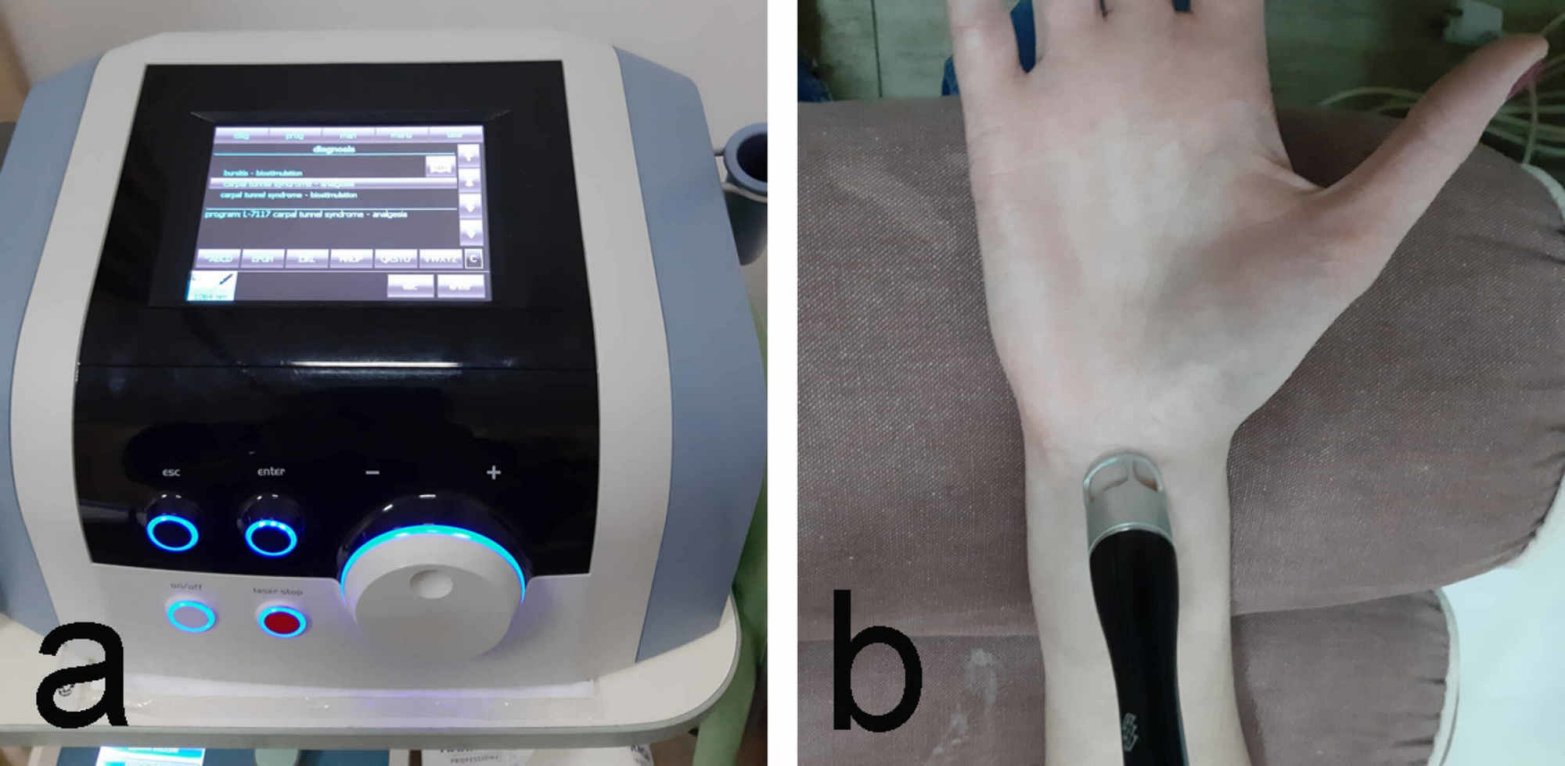
High-intensity laser therapy a: device for high-intensity laser therapy; b: application technique

**Figure 4:**
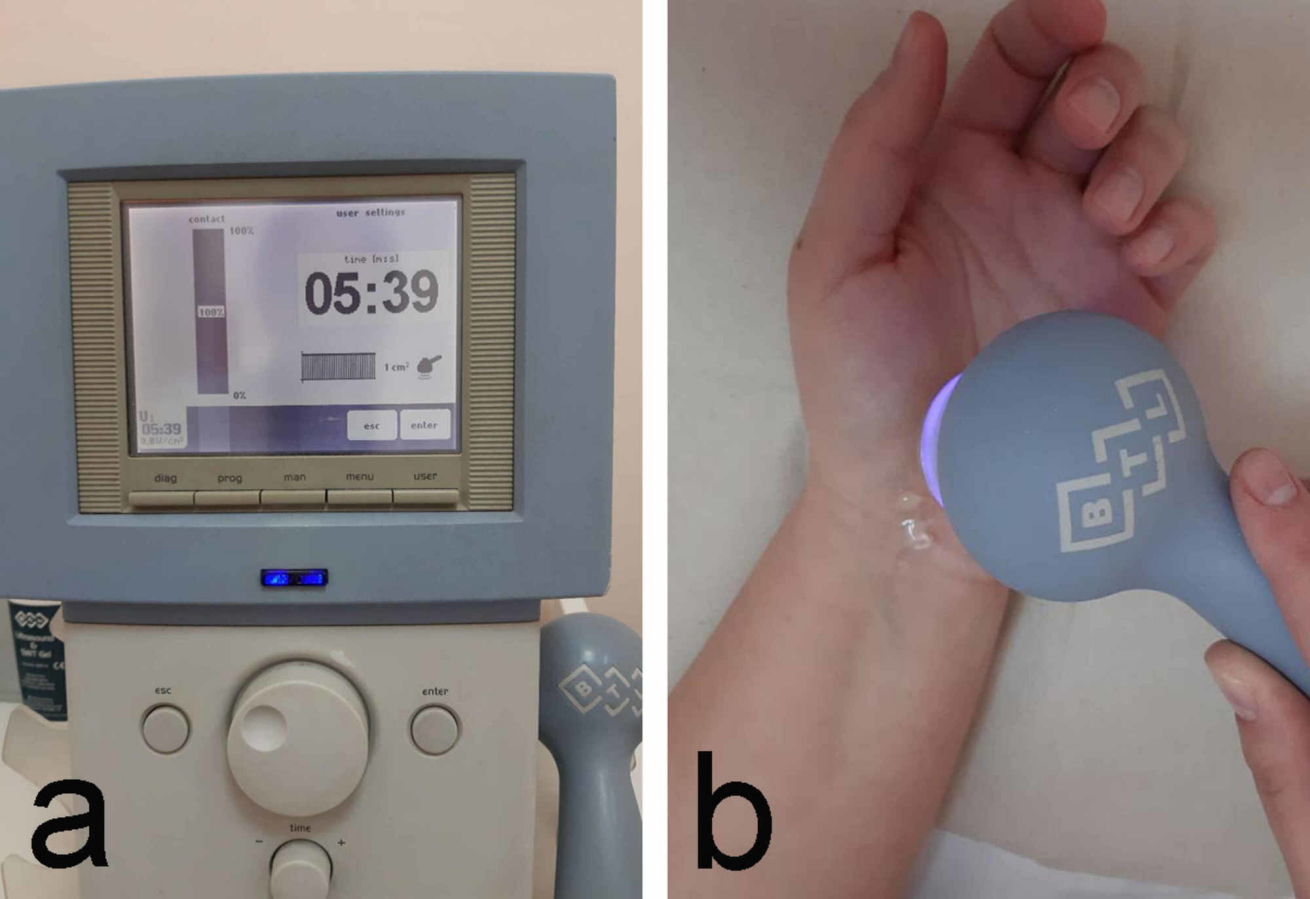
Therapeutic ultrasound a: device; b: application technique

2. Laser therapy is used for symptomatic treatment: for pain and paresthesia [16]. The use of laser therapy in the treatment of CTS is one of the first methods approved by the FDA. The use of both low- and high-intensity laser beams using the corresponding dose regimen is appropriate (Figures 2-3) [17].

3. Ultrasound therapy uses fibrinolytic, anti-inflammatory, and anti-irritant action of the ultrasound. The application is on the projection of the carpal canal using the low-frequency transducer for deeper effect or the high-frequency one for surface effect. The ultrasound intensity is 0.8 to 1.0 W/cm^2^. The duration of the procedure is six minutes. The therapeutic course consists of 10-15 procedures (Figure 4).

When nonsteroidal anti-inflammatory drugs (NSAIDs) are used in the contact gel, the procedure is called ultraphonophoresis; the dosage is lower (0.4-0.6 W/cm^2)^ and the duration is shorter (6-8 minutes). Such a procedure is used frequently after surgical treatment. Other substances, used to treat scars of the skin are preferred in adhesions because of their fibrinolytic effect. According to the Management of CTS in Clinical Practice Guideline by the American Academy of Orthopaedic Surgeons (AAOS), there is evidence of the benefit of ultrasound versus placebo [16]. This study describes the use of 20 procedures for 15 minutes: 1 MHz frequency, 1 W/cm^2^ intensity, five procedures weekly for two weeks, followed by two procedures weekly for another five weeks. Priority is always given to the avoidance of risk to the patient in this therapy. An important contraindication is the presence of acute pain. In such cases, the application of therapeutic ultrasound increased these complaints [16].

**Figure 5:**
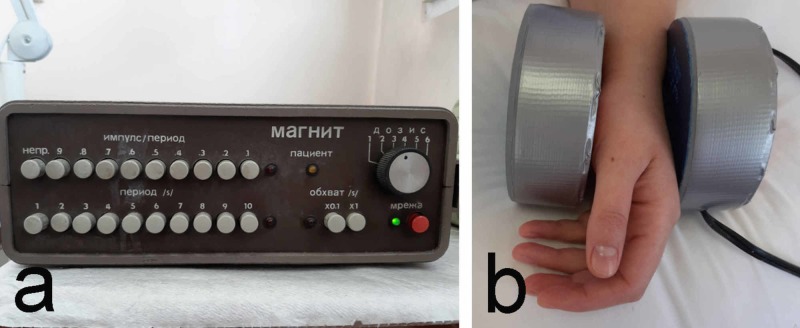
Low-frequency magnetic therapy a: device; b: application technique

4. Magnetotherapy is used to counteract the oxidative action, stimulate oxidative processes and tissue trophic [18]. A low-frequency impulse magnetic field is assigned with parameters of 20-25 mT with period/break ratio 2/8 (Figure 5).

The therapeutic course consists of 10 procedures. Magnetotherapy is contraindicated in patients with pacemakers and is one of the disputed physical factors in the complex treatment of CTS. AAOS does not recommend its use due to a lack of sufficient evidence-based research [16]. Nevertheless, it remains one of the most commonly prescribed physical factors in CTS. Applied before electrophoresis, it significantly increases its therapeutic effect [19]. More and higher-quality research is needed to confirm the positive impact on patients’ main complaints: pain, numbness, and stiffness.

**Figure 6:**
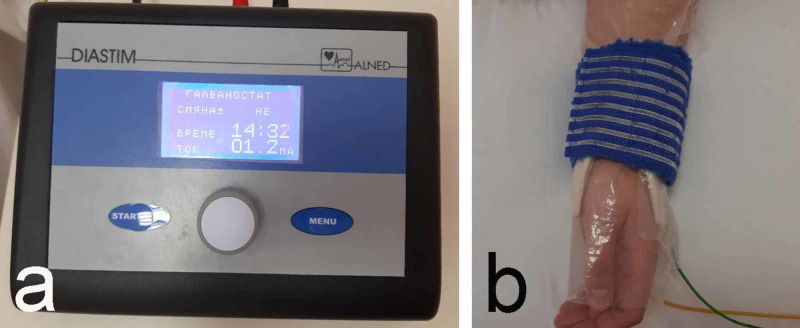
Iontophoresis a: device for galvanization, iontophoresis, and dia-dynamic current; b: application technique

5. Iontophoresis is used for combining the analgesic effect of the galvanic or low-frequency current with the fibrinolytic effect of potassium iodide. The 5% solution of potassium iodide is used for the procedure and is placed on the hydrophile pillow around the negative electrode. The intensity of the current is dosed subjectively (up to 10 mA) to avoid burning and pain sensation. The duration of the procedure is 20 minutes. The therapeutic course consists of 10 procedures (Figure 6).

A positive effect from iontophoresis with galantamine has been described in the literature: improvement of the nerve conduction [20]. However, according to the latest data, galantamine has only proven to be effective in the treatment of Alzheimer's disease [21].

**Figure 7:**
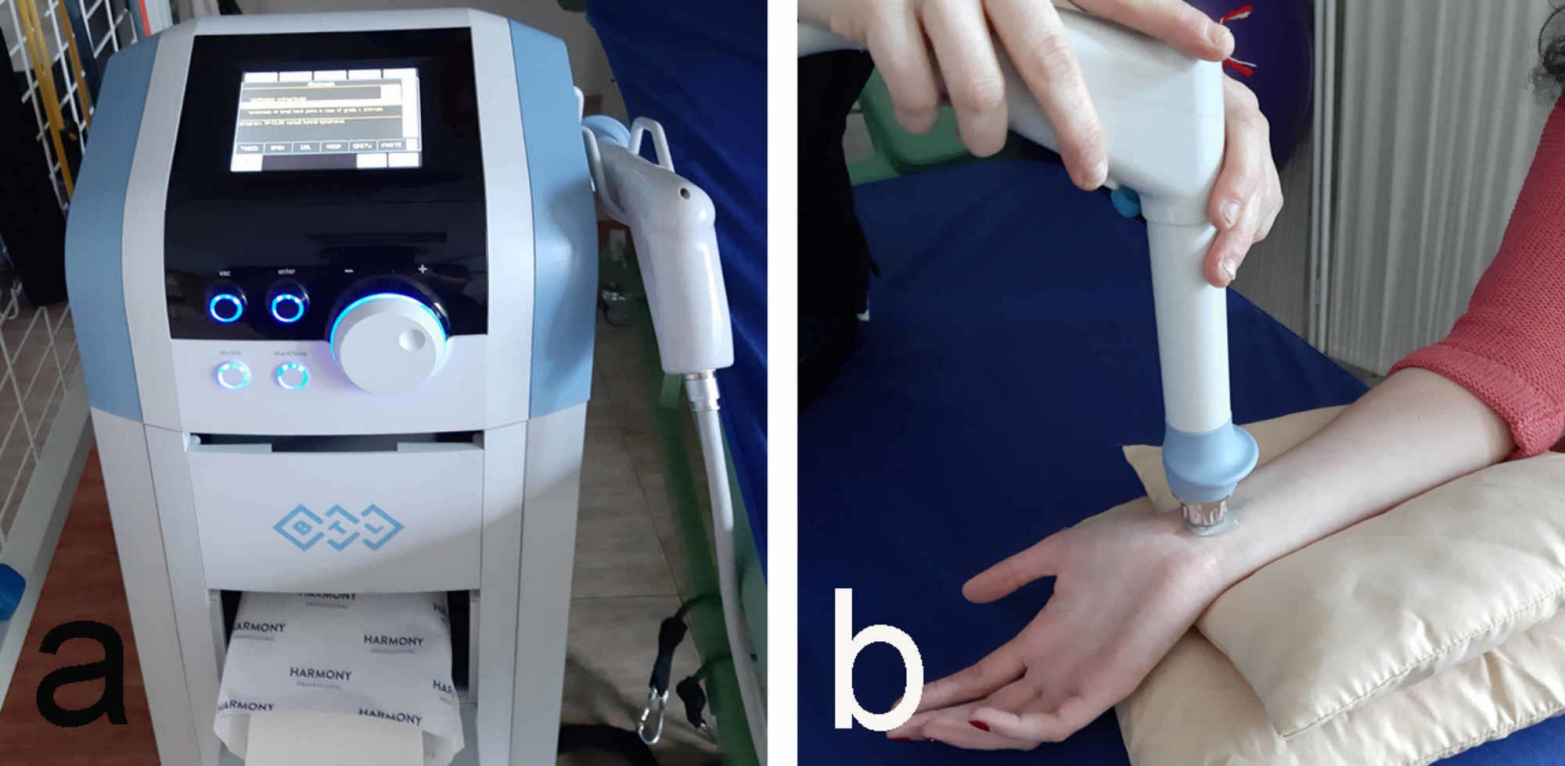
Shockwave therapy a: device; b: application technique

6. Acupuncture can also be used to reduce the pain in CTS. When properly administered, its anesthetic effect is comparable to that of topical corticosteroid administration [22].

7. Shockwave therapy (SWT) is considered to be one of the non-invasive and evidence-based physical approaches to the treatment of CTS [23]. It uses pneumatically generated shock waves with low frequency (5-20 Hz) and pressure of 1-5 bar applied locally in the affected area [24]. SWT is applied in the area of ligamentum carpi transversalis. The therapeutic course consists of 4-6 procedures, with 1-2 procedures per week (Figure 7). It is particularly effective in the early stages of the disease and in young patients where the CTS is associated with occupational overload.

8. Immobilization is recommended in order to eliminate active movements in the affected hand [17]. The wrist is fixed in a neutral position so the tension in the carpal canal will be minimal. The carpometacarpal and the interphalangeal joints are fixed in slight flexion for the same purpose [25,26].

9. Kinesiotherapy, and in particular mechanotherapy, helps in maintaining the trophy of the paretic muscles of the thenar, improving nervous conduction and excitability, and restoring the motor function. Muscle hypotrophy is influenced by a light/attentive massage that should be performed daily [6]. Patients are taught how to perform kinesiotherapy including self-massage at home for a short duration, frequently and with low intensity. Patients should be informed that prolonged and intensive kinesiotherapy and massage are absolutely contraindicated. According to the degree of functional impairment and hypotrophy, an individual kinesiotherapeutic program is developed for the affected hand, varying according to the condition of the patient. It is mandatory to convey instructions on the loading of the elbow and shoulder joint of the eponymous side and of the contralateral limb for prevention. In some patients, despite the properly designed and performed rehabilitation program, the condition does not improve or even deteriorates. This usually is an indication for surgical treatment. In the postoperative period, physiotherapy and rehabilitation again play a key role. A number of authors recommend immobilization for two weeks after surgery [27-31]. Splinting is not a contraindication for active movements in the remaining joints of the upper limb and the contralateral limb. Once the immobilization is over, postoperative rehabilitation is initiated in order to influence the postoperative edema and pain and to prevent the development of fibrosis. The above-described physiotherapeutic factors and methods of kinesiotherapy are used, with emphasis on the active training of the muscles of the affected hand [6].

## Conclusions

CTS is a condition requiring physiotherapy and rehabilitation. Although the CTS is relatively common and causes considerable disability, there is no consensus on the combined use of physical factors in this pathology, and the experts have yet to reach an agreement on the answers to the following questions: 1. How many factors can be used at the same time? 2. How many consecutive courses can be done? 3. How long it is advisable for patients to observe recruited guidelines and recommendations. All practitioners involved in CTS treatment agree that kinesiotherapy needs to be conducted and prophylactic guidelines should be provided for a change in lifestyle. However, this is only in principle, and there are no concrete guidelines pertaining to the frequency, duration, and intensity of the exercises. New studies are constantly emerging in the literature, the results of which often go hand in hand with our observations in clinical practice. Therefore, we recommend that an individual rehabilitation program be created for each patient, according to the clinical picture, the results of the clinical studies, the accompanying diseases, and in view of the professional history. More research is needed comparing different combinations of preformed physical factors as well as suitable frequency, duration, and intensity of the exercises in CTS.
